# Transcription Factors BbPacC and Bbmsn2 Jointly Regulate Oosporein Production in Beauveria bassiana

**DOI:** 10.1128/spectrum.03118-22

**Published:** 2022-11-23

**Authors:** Xi Chen, Wenwen Zhang, Junyao Wang, Shengan Zhu, Xinchi Shen, Hongjun Chen, Yanhua Fan

**Affiliations:** a State Key Laboratory of Silkworm Genome Biology, Biotechnology Research Center, Southwest University, Beibei, People’s Republic of China; Chinese Academy of Sciences

**Keywords:** *Beauveria bassiana*, oosporein, regulatory mechanism, transcription factor

## Abstract

The entomopathogenic fungus Beauveria bassiana can produce the secondary metabolite oosporein under alkaline conditions or in fungus-killed cadavers. However, the regulatory mechanism of oosporein synthesis is not fully understood. In thisstudy, we found that the pH signaling transcription factor BbPacC is involved in the regulation of oosporein production. Overexpression of *BbPacC* promotes oosporein production in B. bassiana at pH 6.0 or under alkaline conditions (pH 8.0), but deletion of this gene abolished oosporein production. Under acidic conditions (pH 4.0), no oosporein production was observed in the wild-type and *BbPacC* overexpression strains. Yeast one-hybrid assays and electrophoretic mobility shift assay (EMSA) confirmed the binding ability of BbPacC with 4 putative PacC-binding sites in the promoter region of *BbOpS3*, a transcription factor located in the oosporein synthetic gene cluster regulating the expression of oosporein synthetic genes. Overexpression of *Bbmsn2*, a previously reported negative regulator of oosporein synthesis, in *OEPacC* or wild-type strains abolished oosporein production in all tested conditions. However, deletion of *Bbmsn2* in the *BbPacC* overexpression strain significantly improved oosporein production even at pH 4.0. These results indicated that BbPacC is a positive regulator of oosporein production and functions jointly with Bbmsn2 to regulate oosporein production in different environments and particularly under alkaline conditions.

**IMPORTANCE** B. bassiana produces the red dibenzoquinone pigment oosporein under certain specific conditions, such as alkaline conditions and fungus-killed cadavers. Ooporein possesses antibiotic and insect immune inhibition activities and plays multiple roles during the infection process of B. bassiana against insect hosts. Several negative regulators involved in oosporein synthesis have been reported; however, we know little about the positive regulators outside the biosynthetic gene cluster. Here, we found that the pH signaling transcription factor BbPacC positively regulates oosporein production by binding to several PacC-binding sites. In addition, our results also indicate that BbPacC jointly acts with the negative regulator Bbmsn2 to regulate oosporein synthesis. Our results provide insight into understanding the regulatory mechanism of oosporein production as well as targets to engineer B. bassiana strains producing high levels of oosporein.

## INTRODUCTION

Entomopathogenic fungi play essential roles in regulating the insect population in nature and have been exploited as fungal biopesticides. After attachment of conidia or other infectious cells on the insect cuticle, entomopathogenic fungi initiate the infection process following germination, penetration of the host cuticle, and proliferation in the host hemolymph. After host death, fungi grow outward (penetrating the cuticle again) and use the insect cadaver as a nutrient source to form mycelium and produce a large number of conidia on the cuticle, which may begin another round of infection under suitable conditions. During this process, varied interactions occur between entomopathogenic fungi and their insect hosts and between these fungi and other saprophytic microorganisms present in the insects, which might be able to feed on the dead insects (1–4). Increasing evidence has suggested that secondary metabolites have important roles in these interactions. Destruxins produced by Metarhizium anisopliae could inhibit host immune reactions, and the loss of synthetic genes of destruxins caused a significant reduction in fungal virulence (5). Similarly, deletion of the beauvericin synthetic gene in Beauveria bassiana also resulted in an approximately 60% reduction in virulence against Galleria mellonella, Helicoverpa zea and Spodoptera exigua (6).

Bacteria in insect cuticles, hemolymph, or gut are also an important factor affecting fungal infection (7–9). In B. bassiana-infected mosquitoes, it was found the opportunistic pathogenic bacterium Serratia marcescens overgrows in the midgut and translocates to the hemocoel, which facilitates fungal killing speed (10). After host death, cadavers are rich in nutrients, not only for fungal but also for bacterial growth. To compete with the bacteria in the cadavers, B. bassiana produces a red dibenzoquinone pigment oosporein after host death to inhibit bacterial growth. Strains with deletion of the oosporein synthetase gene *BbOpS1* cannot efficiently compete for nutrients with bacteria in cadavers and cause less fungal growth and conidiation (11). The transcription factor BbOpS3 in the oosporein cluster (*Opn*) positively regulated the expression of oosporein synthesis genes. Studies have shown that 2 transcription factors, BbSmr1 and Bbmsn2, negatively regulate oosporein production in B. bassiana (11, 12). BbSmr1 is a zinc finger protein involved in the regulation of fungal development and secondary metabolite production. Deletion of *Bbsmr1* resulted in upregulation of the oosporein synthetic gene cluster and then improved oosporein production (11). In addition, the transcription factor Bbmsn2, which regulates the stress responses in B. bassiana, is also a negative regulator of oosporein production. Currently, however, we do not know the regulatory details of oosporein production and are unsure whether there are other proteins involved in the regulatory network.

PacC is a vital component of the Pal/Rim-pH pathway that is responsive to ambient pH changes and regulates the expression of a series of genes, including acid- or alkaline-expressing genes in fungi (13–16). PacC also regulates secondary metabolite production in filamentous fungi. For example, PacC was found to negatively regulate sterigmatocystin (ST) production, and constitutive activation of PacC resulted in 10-fold less production of ST in an Aspergillus nidulans strain (17). In addition, the production of Patulin (PAT) in Penicillium expansum, a mycotoxin that can cause serious health concerns, was also affected by environmental pH (18), and was regulated by PacC. Deletion of *PacC* in P. expansum resulted in the loss of PAT production in conditions with pH values above 6.0, which was caused by the downregulation of 15 genes in the patulin cluster (19). One or more PacC-binding sites were found in the promoters of 9 PAT clusters, indicating that PePacC may directly regulate the expression of PAT synthetic genes. In B. bassiana, one PacC gene was found and functional characterized (20). Deletion of *BbPacC* resulted in no production of oxalic acid and the insecticidal compound dipicolinic acid but promoted the production of a yellow-colored compound bassianolone B (20). Luo et al. reported that wild-type B. bassiana produced more oosporein under alkaline conditions than under acidic conditions (12). We also found 4 PacC-binding sites (T/CGCCAAG) in the promoter of the key transcription factor BbOpS3 in the oosporein cluster. Therefore, we hypothesized that BbPacC might be involved in the regulation of oosporein production. In this study, we constructed BbPacC deletion and overexpression strains, and analyzed oosporein production in these strains to clarify this question. Our results indicated that BbPacC of B. bassiana is a positive regulator of oosporein synthesis and functions jointly with the reported negative regulator Bbmsn2 to control oosporein production under different pH conditions.

## RESULTS

### BbPacC positively regulates oosporein production.

**FIG 1:**
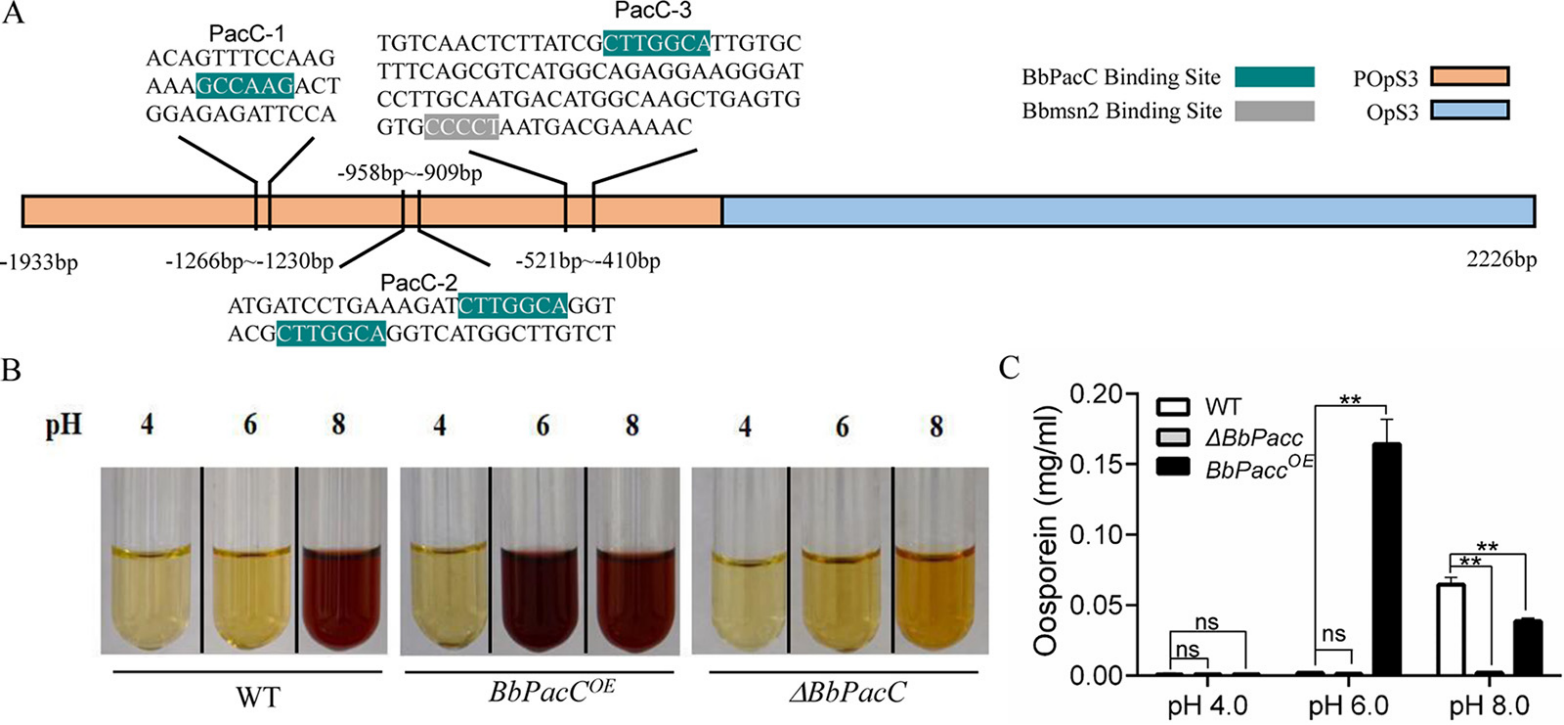
BbPacC positively regulates oosporein production. (A) Analysis of the BbOpS3 promoter. The putative PacC-binding sites were indicated as PacC-1, PacC-2 (contained 2 sites) and PacC-3 sequentially. The potential binding sites of BbPacC and Bbmsn2 are highlighted by the different colors green and gray, respectively. (B) Oosporein production of *BbPacC* deletion and overexpression strains was detected in SDB buffered for different pH values (4, 6 and 8). (C) Oosporein concentration detected by HPLC. **, *P* < 0.01; ns, not significant.

**FIG 2:**
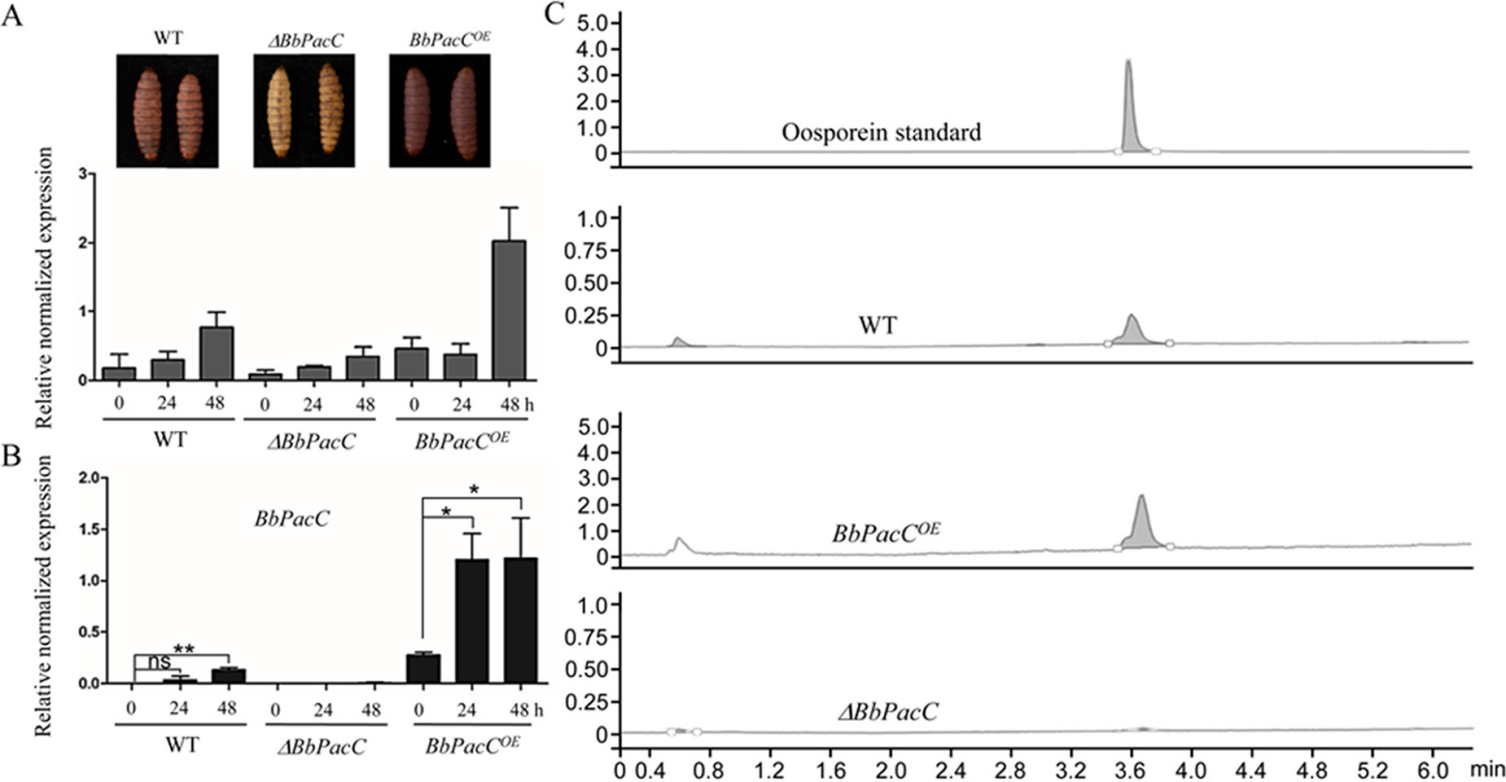
Deletion of *BbPacC* affects oosporein production in G. mellonella cadavers. The expression of *BbOpS3*. (A) and BbPacC (B) was analyzed by RT-PCR after host death. The *Actin* gene was used as the reference gene for normalization. The inset image in A shows the color change of cadavers due to oosporein production. (C) HPLC analysis of oosporein extracted from cadavers killed by *BbPacC* deletion or overexpression strains. *, *P* < 0.05; **, *P* < 0.01; ns, not significant.

BbPacC is an important pH-responsive transcription factor in B. bassiana and is involved in the fungal stress response and the regulation of several secondary metabolites, such as oxalic acid, dipicolinic acid, and bassianolone B (20). BbOpS3 is a GAL4 transcription factor located in the oosporein synthetic gene cluster and regulates the expression of oosporein synthesis genes. To explore the possible regulatory proteins in oosporein production, we analyzed the promoter region of BbOpS3 and found four PacC-binding sites, 3 sites of TGCCAAG (-941 to -937 bp, -930 to -924 bp, -506 to -500 bp) and 1 site of CGCCAAG (-1251 to -1245 bp) (Fig. 1A). To clarify whether BbPacC is involved in oosporein synthesis, we constructed *BbPacC* knockout mutants by replacing the encoding region with the *Sur* resistance gene (Fig. S1A and B). *BbPacC* overexpression strains were also constructed by placing the coding region of *BbPacC* under the control of the constitutive promoter PgpdA from the glyceraldehyde-3-phosphate dehydrogenase gene of B. bassiana (Fig. S1C). The expression levels of *BbPacC* in overexpression strains were detected by real-time PCR (RT-PCR), and 1 strain exhibiting the highest expression level of *BbPacC* (31-fold higher than wild-type) was selected for further study (Fig. S1D). *BbPacC* deletion and overexpression strains were inoculated into SDB with different pH values (4.0, 6.0, and 8.0). After being cultured for 3 to 4 days, the *BbPacC* overexpression strain produced oosporein in media with pH = 6.0 or 8.0 and made the culture medium turn red. However, deletion of *BbPacC* abolished oosporein production in all tested media at pH 4 to 8. The wild-type strain only produced oosporein under alkaline conditions (pH 8.0) (Fig. 1B). High-performance liquid chromatography (HPLC) analysis results confirmed the oosporein-producing conditions, showing concentrations ranging from 0.039 ± 0.001 to 0.166 ± 0.016 mg/mL (Fig. 1C). To further evaluate oosporein production in fungus-killed cadavers, *BbPacC* deletion and overexpression strains were inoculated onto G. menollena larvae. After host death, larvae infected by the wild-type and *BbPacC* overexpression strains appeared red–brown, and HPLC verified oosporein production in the 2 strains. *BbPacC* overexpression strains produced more oosporein than the wild-type strain. After deletion of *BbPacC*, no or less oosporein was produced in fungus-killed larvae (Fig. 2). The expression of *BbOpS3* in fungus-killed cadavers exhibited an almost similar trend to that of *BbPacC*, with the highest expression level achieved in the cadavers where the *BbPacC* overexpression mutant was growing (48 h post-death) (Fig. 2B).

### Interactions between BbPacC and the *BbOpS3* promoter.

**FIG 3:**
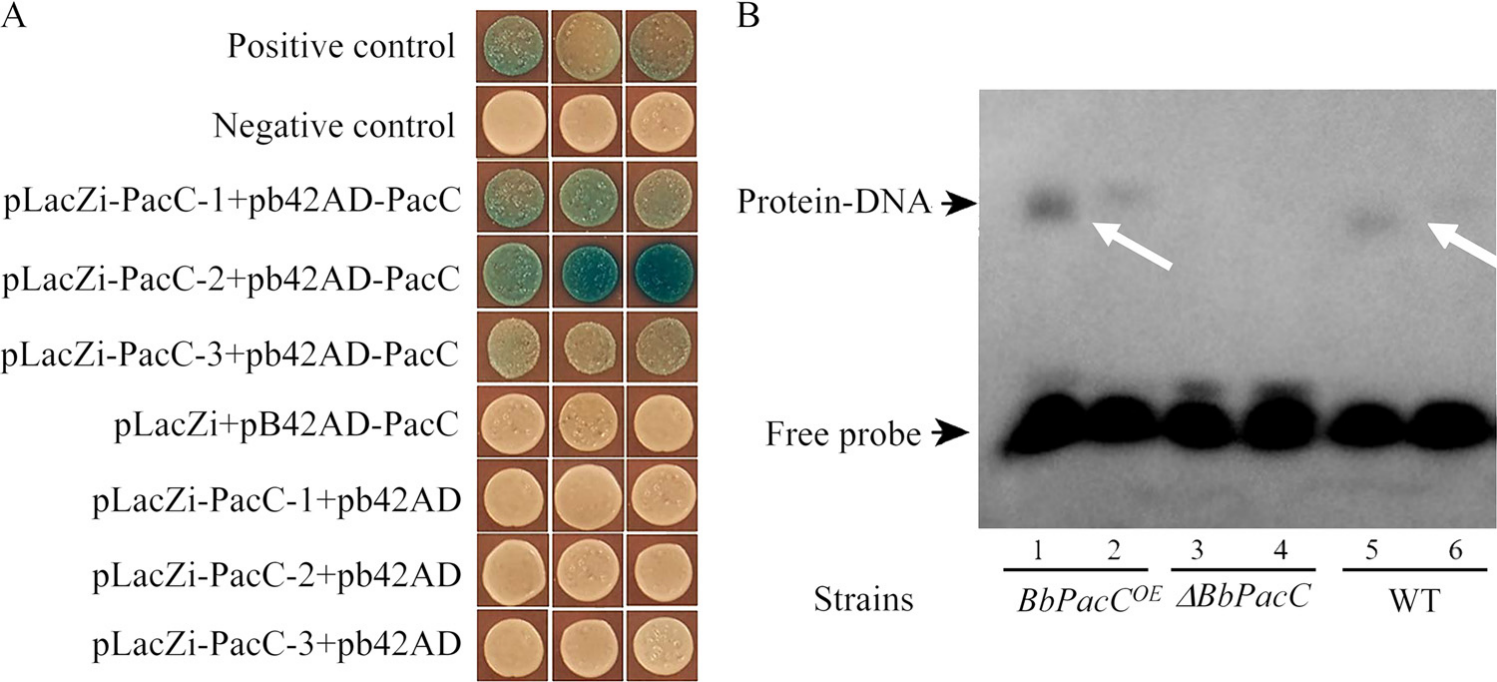
Yeast one-hybrid and EMSA of BbPacC-binding to the *BbOpS3* promoter. (A) Yeast one-hybrid of BbPacC to 4 potential PacC-binding sites in the *BbOpS3* promoter. PacC-binding sited were indicated in Fig. 1A. (B) EMSA of BbPacC bound to a putative PacC site from the *BbPacC* promoter. Proteins were extracted from B. bassiana wild-type, *BbPacC* deletion and overexpression strains and used for EMSA. An unlabeled probe was added in Lanes 2, 4 and 6.

Interactions of BbPacC and predicted PacC-binding sites in the *BbOpS3* promoter were analyzed by yeast one-hybrid and electrophoretic mobility shift assay (EMSA). For yeast one-hybrid assays, 4 predicted BbPacC-binding sites were cloned into 3 vectors. As there is only a 6 bp spacer between the second site (-941 to -937 bp) and the third site (-930 to -924 bp) (Fig. 1A), we designed these 2 sites in 1 fragment, named PacC-2. The results revealed that BbPacC interacts with all 3 tested fragments containing PacC-binding site(s), with fragment 2 (PacC-2), which contains 2 sites exhibiting the strongest binding ability (Fig. 3A). The interaction of BbPacC with the *BbOpS3* promoter was further analyzed by EMSA. Biotin-labeled DNA probes containing the predicted binding site of the *BbOpS3* promoter (-946 bp to -929 bp) were synthesized and incubated with extracted total proteins of fungal strains. A protein-DNA interaction band was observed in the presence of BbPacC protein from *BbPacC* overexpression and wild-type strains. However, for the total proteins from the *BbPacC* deletion strain, no interaction was observed (Fig. 3B).

### *BbPacC* and *Bbmsn2* regulate jointly oosporein production.

**FIG 4:**
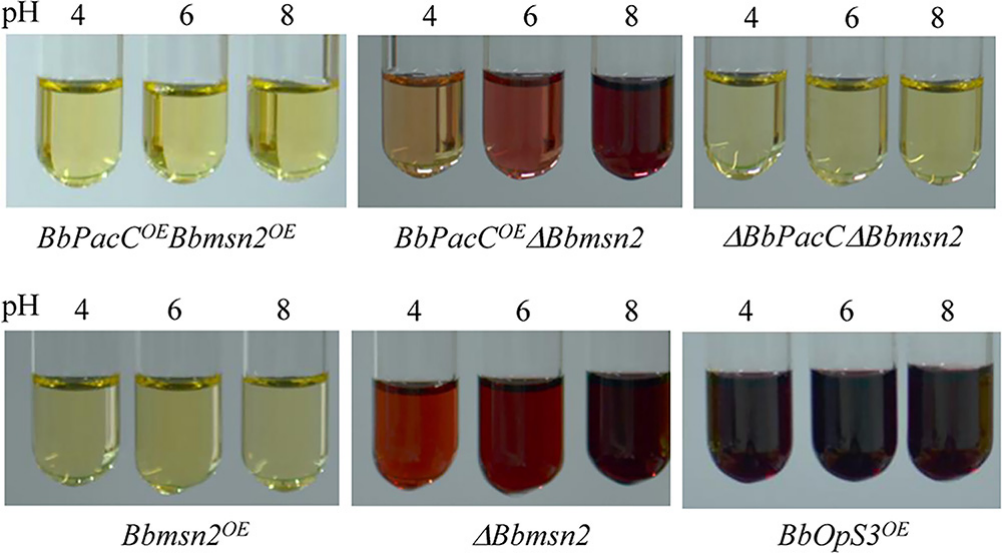
Bbmsn2 and BbPacC jointly regulate oosporein production in B. bassiana. Fungal strains were cultured in SDB buffered for different pH values (4, 6, and 8) for 3 days. The *BbOpS3* overexpression strain (*BbOpS3^OE^*) was used as a positive control.

**FIG 5:**
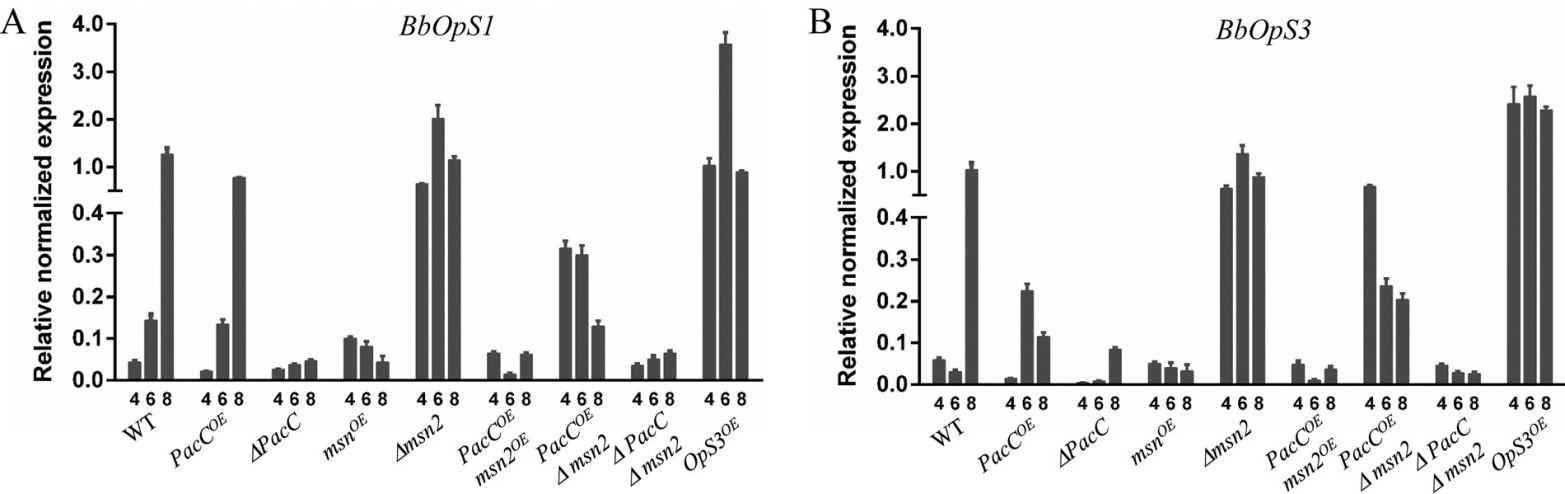
Expression analysis of the oosporein synthetic gene (*BbOpS1*) and the regulatory gene (*BbOpS3*) in various strains. Fungal strains were cultured in SDB (pH 4, 6, and 8) for 3 days, and total RNA was extracted for RT-PCR analysis. The *Actin* gene was used as the reference gene for normalization.

*Bbmsn2* has been shown to negatively regulate oosporein production in B. bassiana (12). To test whether *Bbmsn2* and *BbPacC* regulate jointly oosporein production, *BbPacC* was overexpressed or deleted in *Bbmsn2* strains, including *ΔBbmsn2* and *OEBbmsn2*. Firstly, we constructed *ΔBbmsn2* and *OEBbmsn2* strains by replacing the encoding region with the *Sur* resistance gene or placing the encoding region of *Bbmsn2* under the control of a constitutive promoter Pb3 from the glyceraldehyde-3-phosphate dehydrogenase gene of B. bassiana (Fig. S2A and B). Then, we overexpressed or deleted *BbPacC* in *ΔBbmsn2* and *OEBbmsn2* strains, obtaining the strains *OEBbPacCOEBbmsn2*, *OEBbPacCΔBbmsn2*, and *ΔBbPacCΔBbmsn2*. The expression levels of *BbPacC* and *Bbmsn2* in different overexpression and knockout strains were detected by RT-PCR, and some strains exhibiting the highest expression level of *BbPacC* was selected for further study, as well as verification of the knockout gene (Fig. S2G). These strains were cultured in SDB buffer with various pH values for 4 days, and oosporein production was detected. Although the expression of *BbPacC* promoted oosporein production at pH 6.0 and 8.0, overexpression of *Bbmsn2* completely inhibited oosporein production under these tested conditions regardless of whether BbPacC is overexpressed (Fig. 4). Conversely, deletion of *Bbmsn2* in the *BbPacC* overexpression strain further improved oosporein synthesis even at pH 4.0, in which wild-type and *OEBbPacC* strains do not synthesize oosporein (Fig. 4). These results were similar with that of Luo et al., in which *Bbmsn2* mutant produced oopsorein under pH 5.1 to 7.4 in SDB (12). In double deletion mutants of *BbPacC* and *Bbmsn2*, no oosporein production was observed in media with various pH values. The oosporein production of the *OEBbOps3* strain is also presented. Oosporein was produced under 3 pH conditions, and the content increased from pH 4.0 to pH 8.0. The expression levels of the polyketone synthetase genes *BbOpS1* and *BbOps3* exhibited a positive relationship with the oosporein production in these tested strains (Fig. 5). These results indicated that BbPacC and Bbmsn2 play positive and negative regulatory roles, respectively, in oosporein production and jointly regulate oosporein synthesis in various environments.

### BbPacC and Bbmsn2 exhibited different expression models under various pH values.

**FIG 6:**
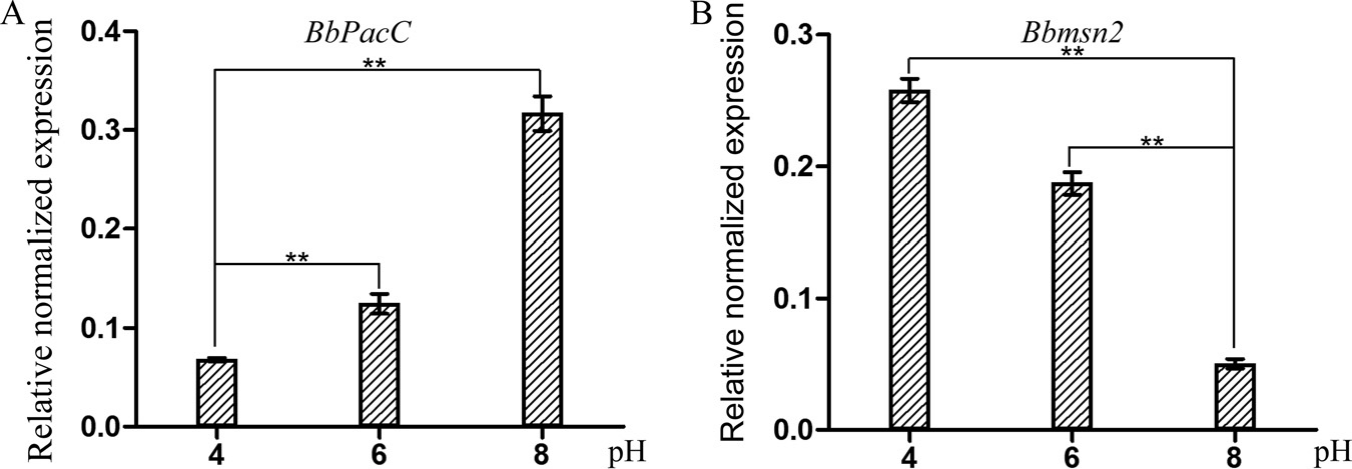
Expression of *BbPacC* and *Bbmsn2* in *B.* bassiana under different pH values. Wild-type *B.* bassiana was cultured in SDB buffered for various pH values (4, 6, and 8) for 3 days. Total RNA was extracted, and RT-PCR was performed as described in Materials and Methods. The *Actin* gene was used as the reference gene for normalization. **, *P* < 0.01.

It has been reported that alkaline conditions can promote oosporein production, but acidic conditions inhibit oosporein synthesis (12). To understand the possible mechanism regulating oosporein production under various pH conditions, we analyzed the expression levels of *BbPacC* and *Bbmsn2* in the wild-type strain in SDB buffered for different pH values. *BbPacC* was expressed at the highest level at pH 8.0 but the lowest at pH 4.0; however, a converse expression pattern of *Bbmsn2* was observed, with the highest expression at pH 4.0. High-level expression of *Bbmsn2* at pH 4.0 explains why the *BbPacC* overexpression strain cannot synthesize oosporein under this condition (Fig. 6).

## DISCUSSION

Oosporein is reported in Oospora colorans, Beauveria brongniartii and some soil/endophytic fungi (21–23). Based on the genomic DNA sequence, the oosporein synthetic gene cluster was also identified in the B. bassiana genome, and the biosynthetic pathway and biological functions were verified experimentally (24–26). Two negative regulators of oosporein production, BbSmr1 and Bbmsn2, have been reported (8, 9), and our results showed that the transcription factor BbPacC positively regulates oosporein production. Overexpression of BbPacC significantly improved oosporein production in SDB under pH 6 or 8; however, deletion of *BbPacC* severely impaired oosporein synthesis. In addition, our results showed that BbPacC functions jointly with the negative regulator Bbmsn2 to affect oosporein synthesis under various pH conditions. This provides insight into the regulatory mechanism of oosporein synthesis.

The production of fungal secondary metabolites is impaired by different environmental signals, such as nutrients, temperature, light, pH, and stressors (27). Therefore, transcription factors involved in nutrient source, light intensity, redox status, and ambient pH values, such as LaeA, AreA, HapX, and PacC, are responsible for the regulation of the production of some secondary metabolites (28). In entomopathogenic fungi, PacC is involved in acid/alkaline responses, many physiological processes, and mycelial growth, stress response, virulence, and secondary metabolite production (20, 29). Deleting *PacC* (*MrPacC*) from Metarhizium robertsii reduced fungal tolerance to salt/metal challenges and full virulence and downregulated the expression of chitinase and glucosyltransferase genes (29). In B. bassiana, *BbPacC* regulates not only fungal responses to various stressors, including osmotic stress and SDS, but also the production of oxalic acid, dipcolinic acid, and bassianoline B (20). In this study, BbPacC was also found to regulate oosporein production. It is worth noting that orsellinic acid is the intermediate product of the oosporein synthesis pathway produced by the polyketide synthetase BbOpS1 in the oosporein cluster. These results indicated BbPacC plays important roles in the production of acid substances in B. bassiana and we may explore more secondary metabolites with BbPacC deletion or overexpression strains.

BbOpS3 is a Gal4-like transcription factor in the oosporein synthesis gene cluster. Overexpression of *BbOpS3* can constitutively activate oosporein, but the *BbOpS3* mutant caused no oosporein production, indicating that BbOpS3 directly regulates oosporein production (26). Therefore, the regulatory mechanism of BbOpS3 expression is the key to understanding oosporein synthesis. Our results showed that BbPacC can bind the putative PacC sites in the promoter of *BbOpS3*, indicating that BbPacC may directly regulate the expression of *BbOpS3*. Deletion of *BbPacC* abolished oosporein production, further verifying that BbPacC is a positive regulator in oosporein production. However, it is unclear whether other positive regulatory factors in B. bassiana control oosporein production.

Studies have shown that the production of oosporein in artificial media is not consistent and greatly depends on nutrient compositions and pH values. A study found that only a small fraction (5 out of 16) of B. bassiana strains freshly isolated from cadavers could produce oosporein under the tested conditions, and serial passage on artificial media caused the oosporein-ng strains to lose oosporein-producing ability (30). Therefore, there may be a complex regulatory cascade in oosporein synthesis in B. bassiana. Although BbPacC is a positive regulator of oosporein production, overexpression of *BbPacC* did not lead to oosporein production at pH 4.0. Deleting another transcription factor, Bbmsn2, from the *OEBbPacC* strain resulted in oosporein synthesis at pH 4.0, indicating that, at low pH Bbmsn2 blocked oosporein synthesis even with high levels of *BbPacC*. RT–PCR analysis showed that *Bbmsn2* has a higher expression level under acidic conditions in the B. bassiana wild-type strain, but the expression of *BbPacC* exhibits the opposite trend: alkaline > neutral > acidic conditions, which also explains why B. bassiana produces oosporein in alkaline environments. In the *BbOpS3* promoter, there is also a putative msn2-binding site AGGGG (-436 to -500 bp). We have tried to analyze the interaction between Bbmsn2 and the putative binding site. However, no evident interaction was observed (data not shown). Currently, we are not sure whether the regulatory mechanism of Bbmsn2 on *BbOpS3* expression is direct or indirect. In a previous study, we found that the transcription factor BbSmr1 also negatively regulates oosporein production. However, the relationship among these transcriptional factors needs further study. Combining these results, we hypothesize that there is a complex regulatory cascade for oosporein production in which more regulatory proteins, except those examined here, may be involved. Further studies will clarify the detailed regulatory mechanism of oosporein synthesis, which will aid in understanding the biological roles of oosporein in B. bassiana.

## MATERIALS AND METHODS

### Strains and culture conditions.

B. bassiana strain *Bb0062* (CGMCC 7.34) was used in this study and typically cultured in Czapek-Dox Broth and Czapek-Dox Agar (CZB)/CZA, Potato Dextrose Broth (PDB)/PDA, or Sabouraud Dextrose Broth (SDB)/SDA. Escherichia coli DH5α and Agrobacterium tumefaciens AGL-1 were used for routine DNA manipulation and fungal transformation, respectively.

### Construction of deletion and overexpression strains.

The primers used for DNA manipulation are listed in Table S1. To construct the *BbPacC* deletion mutant, a 651 bp sequence in the *BbPacC* ORF was replaced with the *Sur* gene cassette (1694 bp). The upstream (952 bp) and downstream (1510 bp) regions were amplified by PCR using genomic DNA as a template and cloned into the EcoRI/SpeI and XbaI/HindIII sites of the pK2-bar vector. The resultant vector was verified by sequencing and transformed into A. tumefaciens. B. bassiana transformation mediated by A. tumefaciens was performed as described (31). Correct mutants were screened by PCR using the primers PacC-t1/t2 and subsequently verified by RT–PCR. Using the same method for the *BbPacC* mutant, the *Bbmsn2* mutant was constructed by replacing a 464 bp fragment with this gene with the *Sur* gene cassette (2560 bp). The upstream (1532 bp) and downstream (1230 bp) regions of the *Bbmsn2* deletion region were amplified and cloned into pK2-Sur. The obtained *Bbmsn2* deletion construct was transformed into B. bassiana wild-type and *ΔBbPacC* strains, and the *Bbmsn2* mutant (*ΔBbmsn2*) and double deletion mutant (*ΔBbmsn2ΔBbPacC*) were obtained.

To construct *the BbPacC* overexpression vector, the ORF was amplified with the primers Pacc-O1/O2 using genomic DNA as a template. The BbPacC fragment was cloned downstream of the constitutive promoter *PgpdA* in the pK2-bar-PgpdA vector using a seamless cloning kit (ClonExpress, Vazyme). *BbPacC* overexpression strains were verified by PCR using the primers pgpdA-up, located in the promoter, and pgpdA and PacCcDNA2.

### Oosporein extraction and detection.

Fungal strains were cultured in 0.5 × SDB buffered with 0.2 M Na_2_PO_4_-0.1 M citric acid to pH = 4 and 6, and 0.05 M glycine-0.05 M NaOH to pH = 8, respectively. Fungal cultures were grown at 26°C for 3 days, and oosporein was extracted and detected by HPLC as described (11). HPLC was performed with an Agilent ZORBAX SB-C18 column (25 cm × 4.6 mm, 5 μm) with a formic acid (0.1‰)/acetonitrile gradient consisting of 5% acetonitrile (0 to 3 min), 5 to 100% acetonitrile (3 to 18 min), and 100% acetonitrile (18 to 21 min). Purified oosporein was used to prepare a standard curve.

### RT-PCR analysis.

Total RNA was extracted from various strains after being cultured in 0.5 × SDB for 3 days or from fungus-killed G. mellonella larvae. Real-time PCR was performed following the previously described method (11), with the indicated primers in Table S1. The relative transcript levels of target genes were normalized to *actin* (GenBank accession no. HQ232398).

### Fungal infection.

Fungal strains were grown on PDA plates for 2 weeks, and conidial suspensions were prepared with 0.05% Tween 80 and adjusted to 10^7^ conidia/mL. Fourth instar G. mellonella larvae were immersed in conidial suspension for 15 s and were kept in Petri dishes (ϕ = 150 mm). After the fungi killed the larvae, cadavers were transferred into a new Petri dish, and oosporein production was observed by a color change and HPLC analysis.

### Yeast one-hybrid assay.

To perform a yeast one-hybrid assay, the *BbPacC* ORF (2004 bp) was amplified with the primer pair *BbPacC*-AD42-F/R using B. bassiana cDNA as a template and cloned into pB42AD to obtain pB42AD-BbPacC (effector). PacC-binding sites containing 3-bp of its upstream and downstream sequences respectively were repeated two or three times, and designed into a primer pair (Table S1). As there is only 6-bp space between second and third sites, they were designed into one fragment and repeated two times (Fig. 1A). Primers (10 μm) were treated by polynucleotide kinase and used to form double stranded DNA with EcoRI and XhoI sites at both ends, respectively. The obtained fragments were cloned into the pLacZi vectors to construct reporter vectors. The effector and reporters were co-transferred into the *EGY48* yeast strain to confirm interactions in yeast cells. Transformants were grown on SD/-Ura/-Trp medium at 30°C for 3 to 5 days, and positive colonies were transferred onto SD/Gal/Raf medium supplemented with X-gal (80 mg/L) for color development, with empty pB42AD and pLacZi serving as negative controls.

### Fungal protein extraction and EMSA analysis.

B. bassiana wild-type, *BbPacC^OE^*, and *ΔBbPacC* strains were cultured in 0.5 × SDB (pH = 8) for 4 days. Fungal proteins were extracted from mycelium using a filamentous fungal protein extraction kit (BestBio, China). EMSA was performed using the LightShift Chemiluminescent EMSA kit (Thermo Fischer Scientific) following the manufacturer’s instructions. The biotin-labeled DNA fragment containing the PacC-binding site in the *BbOpS3* promoter (-946 bp to -929 bp) was synthesized (Huada Biological Technology). In competition assays, a 200-fold unlabeled probe was added.

### Data analyses.

Experiments were performed in triplicate and repeated three times. Gene expression analyses oosporein concentration were graphed using GraphPad prism 7.0 software (GraphPad) and significance was assessed with one-way ANOVA. Asterisks were used to denote significance (*, *P* < 0.05; **, *P* < 0.01).

## References

[1] Pedrini N, Ortiz-Urquiza A, Huarte-Bonnet C, Fan Y, Juarez MP, Keyhani NO. 2015. Tenebrionid secretions and a fungal benzoquinone oxidoreductase form competing components of an arms race between a host and pathogen. Proc Natl Acad Sci USA 112:E3651–E3660. doi:10.1073/pnas.1504552112. 26056261 PMC4507192

[2] Kim T, Kim YJ. 2005. Overview of innate immunity in *Drosophila*. J Biochem Mol Biol 38:121–127. doi:10.5483/bmbrep.2005.38.2.121. 15826489

[3] Sosa-Gomez DR, Boucias DG, Nation JL. 1997. Attachment of *Metarhizium anisopliae* to the southern green stink bug *Nezara viridula* cuticle and fungistatic effect of cuticular lipids and aldehydes. J Invertebr Pathol 69:31–39. doi:10.1006/jipa.1996.4619. 9028925

[4] Ortiz-Urquiza A, Keyhani NO. 2013. Action on the surface: fntomopathogenic fungi versus the insect cuticle. Insects 4:357–374. doi:10.3390/insects4030357. 26462424 PMC4553469

[5] Wang B, Kang Q, Lu Y, Bai L, Wang C. 2012. Unveiling the biosynthetic puzzle of destruxins in *Metarhizium* species. Proc Natl Acad Sci USA 109:1287–1292. doi:10.1073/pnas.1115983109. 22232661 PMC3268274

[6] Xu Y, Orozco R, Kithsiri Wijeratne EM, Espinosa-Artiles P, Leslie Gunatilaka AA, Patricia Stock S, Molnar I. 2009. Biosynthesis of the cyclooligomer depsipeptide bassianolide, an insecticidal virulence factor of *Beauveria bassiana*. Fungal Genet Biol 46:353–364. doi:10.1016/j.fgb.2009.03.001. 19285149

[7] Boucias DG, Zhou Y, Huang S, Keyhani NO. 2018. Microbiota in insect fungal pathology. Appl Microbiol Biotechnol 102:5873–5888. doi:10.1007/s00253-018-9089-z. 29802479

[8] Kikuchi Y. 2009. Endosymbiotic bacteria in insects: their diversity and culturability. Microbes Environ 24:195–204. doi:10.1264/jsme2.me09140s. 21566374

[9] Toledo AV, Alippi AM, de Remes Lenicov AM. 2011. Growth inhibition of *Beauveria bassiana* by bacteria isolated from the cuticular surface of the corn leafhopper, *Dalbulus maidis* and the planthopper, *Delphacodes kuscheli*, two important vectors of maize pathogens. J Insect Sci 11:1–13. doi:10.1673/031.011.0129. 21529147 PMC3281398

[10] Wei G, Lai Y, Wang G, Chen H, Li F, Wang S. 2017. Insect pathogenic fungus interacts with the gut microbiota to accelerate mosquito mortality. Proc Natl Acad Sci USA 114:5994–5999. doi:10.1073/pnas.1703546114. 28533370 PMC5468619

[11] Fan Y, Liu X, Keyhani NO, Tang G, Pei Y, Zhang W, Tong S. 2017. Regulatory cascade and biological activity of *Beauveria bassiana* oosporein that limits bacterial growth after host death. Proc Natl Acad Sci USA 114:E1578–E1586. doi:10.1073/pnas.1616543114. 28193896 PMC5338512

[12] Luo Z, Li Y, Mousa J, Bruner S, Zhang Y, Pei Y, Keyhani NO. 2015. *Bbmsn2* acts as a pH-dependent negative regulator of secondary metabolite production in the entomopathogenic fungus *Beauveria bassiana*. Environ Microbiol 17:1189–1202. doi:10.1111/1462-2920.12542. 24965521

[13] Penalva MA, Tilburn J, Bignell E, Arst HN, Jr. 2008. Ambient pH gene regulation in fungi: making connections. Trends Microbiol 16:291–300. doi:10.1016/j.tim.2008.03.006. 18457952

[14] Rascle C, Dieryckx C, Dupuy JW, Muszkieta L, Souibgui E, Droux M, Bruel C, Girard V, Poussereau N. 2018. The pH regulator PacC: a host-dependent virulence factor in *Botrytis cinerea*. Environ Microbiol Rep 10:555–568. doi:10.1111/1758-2229.12663. 30066486

[15] Trushina N, Levin M, Mukherjee PK, Horwitz BA. 2013. PacC and pH-dependent transcriptome of the mycotrophic fungus *Trichoderma virens*. BMC Genomics 14:138. doi:10.1186/1471-2164-14-138. 23445374 PMC3618310

[16] Martins MP, Martinez-Rossi NM, Sanches PR, Gomes EV, Bertolini MC, Pedersoli WR, Silva RN, Rossi A. 2019. The pH signaling transcription factor PAC-3 regulates metabolic and developmental processes in pathogenic fungi. Front Microbiol 10:2076. doi:10.3389/fmicb.2019.02076. 31551996 PMC6738131

[17] Keller NP, Nesbitt C, Sarr B, Phillips TD, Burow GB. 1997. pH regulation of sterigmatocystin and aflatoxin biosynthesis in *Aspergillus spp*. Phytopathology 87:643–648. doi:10.1094/PHYTO.1997.87.6.643. 18945083

[18] Zong Y, Li B, Tian S. 2015. Effects of carbon, nitrogen and ambient pH on patulin production and related gene expression in *Penicillium expansum*. Int J Food Microbiol 206:102–108. doi:10.1016/j.ijfoodmicro.2015.05.007. 26001378

[19] Chen Y, Li B, Xu X, Zhang Z, Tian S. 2018. The pH-responsive PacC transcription factor plays pivotal roles in virulence and patulin biosynthesis in *Penicillium expansum*. Environ Microbiol 20:4063–4078. doi:10.1111/1462-2920.14453. 30370586

[20] Luo Z, Ren H, Mousa JJ, Rangel DE, Zhang Y, Bruner SD, Keyhani NO. 2017. The PacC transcription factor regulates secondary metabolite production and stress response, but has only minor effects on virulence in the insect pathogenic fungus *Beauveria bassiana*. Environ Microbiol 19:788–802. doi:10.1111/1462-2920.13648. 28083986

[21] Eyal J, Mabud MA, Fischbein K, Walter J, Osborne L, Landa Z. 1994. Assessment of *Beauveria bassiana* Nov. EO-1 strain, which produces a red pigment for microbial control. Appl Biochem Biotechnol 44:65–80. doi:10.1007/BF02921852.

[22] Kogl F, Van Wessem G. 1944. Analysis concerning pigments of fungi XIV: concerning oosporein, the pigment of *Oospora colorans* van Beyma. Recl Trav Chim Pays Bas 63:5–24. doi:10.1002/recl.19440630102. (In German).

[23] Strasser H, Abendstein D, Stuppner H, Butt TM. 2000. Monitoring the distribution of secondary metabolites produced by the entomogenous fungus *Beauveria brongniartii* with particular reference to oosporein. Mycol Res 104:1227–1233. doi:10.1017/S0953756200002963.

[24] Xiao G, Ying SH, Zheng P, Wang ZL, Zhang S, Xie XQ, Shang Y, St Leger RJ, Zhao GP, Wang C, Feng MG. 2012. Genomic perspectives on the evolution of fungal entomopathogenicity in *Beauveria bassiana*. Sci Rep 2:483. doi:10.1038/srep00483. 22761991 PMC3387728

[25] Gibson DM, Donzelli BG, Krasnoff SB, Keyhani NO. 2014. Discovering the secondary metabolite potential encoded within entomopathogenic fungi. Nat Prod Rep 31:1287–1305. doi:10.1039/c4np00054d. 25148015

[26] Feng P, Shang Y, Cen K, Wang C. 2015. Fungal biosynthesis of the bibenzoquinone oosporein to evade insect immunity. Proc Natl Acad Sci USA 112:11365–11370. doi:10.1073/pnas.1503200112. 26305932 PMC4568701

[27] Keller NP. 2019. Fungal secondary metabolism: regulation, function and drug discovery. Nat Rev Microbiol 17:167–180. doi:10.1038/s41579-018-0121-1. 30531948 PMC6381595

[28] Brakhage AA. 2013. Regulation of fungal secondary metabolism. Nat Rev Microbiol 11:21–32. doi:10.1038/nrmicro2916. 23178386

[29] Huang W, Shang Y, Chen P, Gao Q, Wang C. 2015. MrpacC regulates sporulation, insect cuticle penetration and immune evasion in *Metarhizium robertsii*. Environ Microbiol 17:994–1008. doi:10.1111/1462-2920.12451. 24612440

[30] Basyouni SHE, Brewer D, Vining L. 1968. Pigments of the genus *Beauveria*. Can J Bot 46:441–448. doi:10.1139/b68-067.

[31] Fang W, Zhang Y, Yang X, Zheng X, Duan H, Li Y, Pei Y. 2004. *Agrobacterium tumefaciens*-mediated transformation of *Beauveria bassiana* using an herbicide resistance gene as a selection marker. J Invertebr Pathol 85:18–24. doi:10.1016/j.jip.2003.12.003. 14992856

