# Supplementary material for: Transcription Factors BbPacC and Bbmsn2 Jointly Regulate Oosporein Production in Beauveria bassiana
Source: Microbiol Spectr. 2022 Nov 23;10(6):e03118-22. doi: 10.1128/spectrum.03118-22 (PMC9769838; doi:10.1128/spectrum.03118-22)
Supplement: Supplemental file 1 — Supplemental material. Download spectrum.03118-22-s0001.pdf, PDF file, 0.3 MB [file spectrum.03118-22-s0001.pdf]

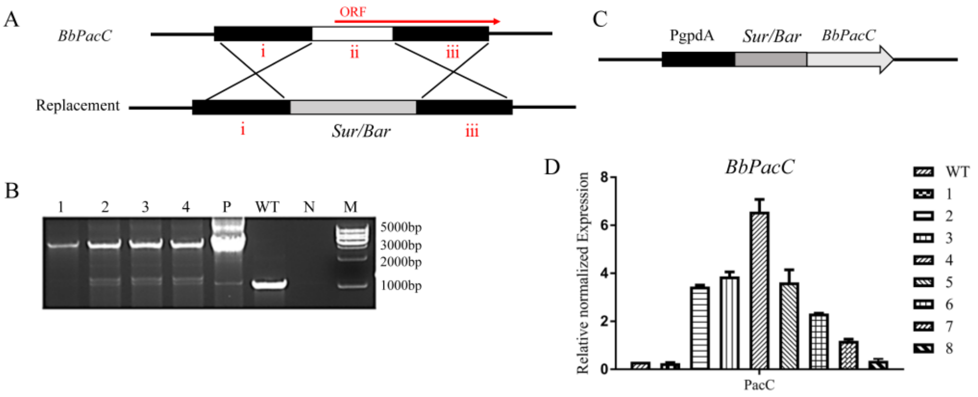

Fig S1. Screening of *ΔBbPacC* and *BbPacC*^{OE} strains. (A) Schematic of construction of *BbPacC* mutants. *Sur/Bar*, the chlorimuron-ethyl or the glyphosate resistance gene. ⅰ, upstream fragment of the deletion construction (1500 bp). ⅱ, deletion region (200 bp). ⅲ, downstream fragment of the deletion construction (1300 bp). (B) Confirmation of *BbPacC* knockout strains by PCR. Lane M, Marker 5000. lane 1-4, four *BbPacC* mutants. P, positive control with knockout vector as template. N, negative control with H_2O as template. (C), Schematic of *BbPacC* overexpression construction. PgpdA, glyceraldehyde-3-phosphate dehydrogenase promoter from *B. bassiana*. (D), Real-time PCR analysis of *BbPacC* overexpression strains (1-8).

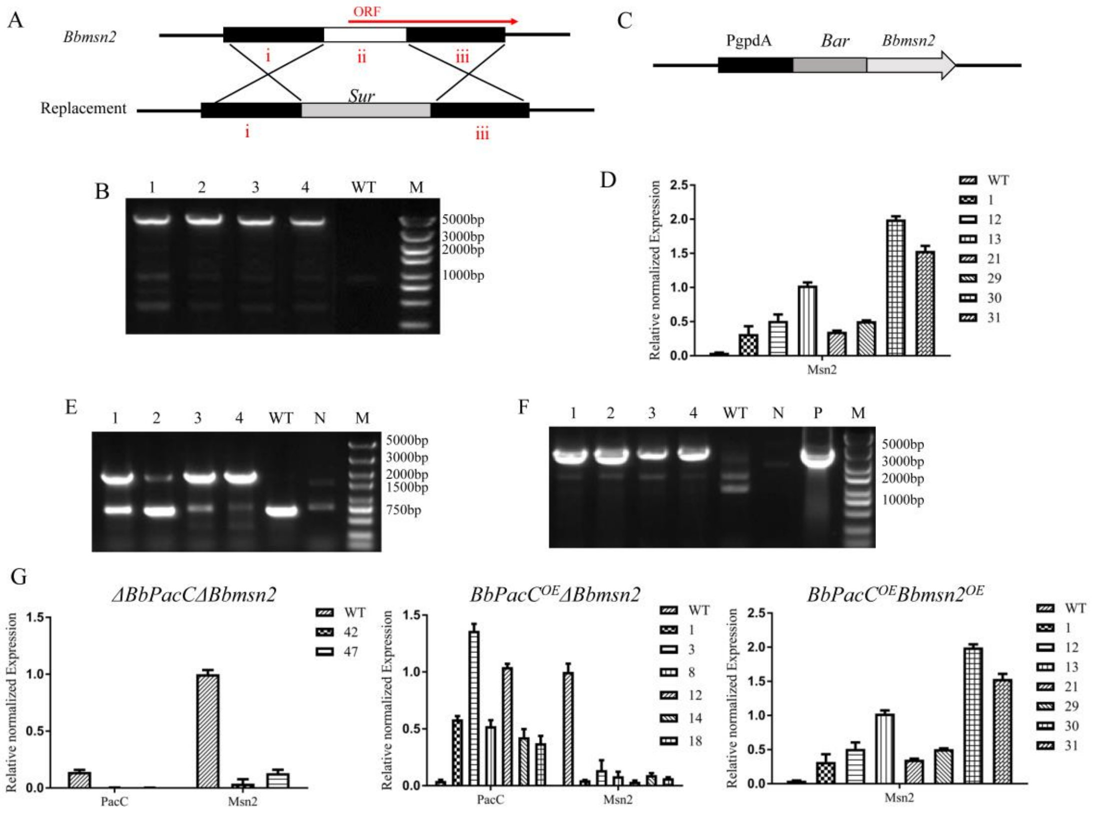

Fig S2. Screening of *ΔBbmsn2* and $Bbmsn2^{OE}$, $PacC^{OE}msn2^{OE}$, $PacC^{OE}\Delta msn2$, *ΔPacCΔmsn2* strains. (A) Schematic of construction of *Bbmsn2* mutants. *Sur*, the chlorimuron-ethyl. ⅰ, upstream fragment of the deletion construction (1300 bp). ⅱ, deletion region (250 bp). ⅲ, downstream fragment of the deletion construction (1400 bp). (B) Confirmation of *Bbmsn2* knockout strains by PCR. Lane M, Marker 5000. lane 1-4, four *Bbmsn2* mutants. (C) Schematic of *Bbmsn2* overexpression construction. PgpdA: glyceraldehyde-3-phosphate dehydrogenase promoter from *B. bassiana*, *Bar*: the glyphosate resistance gene. (D) Real-time PCR analysis of *Bbmsn2* overexpression strains. (E, F) Confirmation of *ΔBbPacCΔBbmsn2*, $BbPacC^{OE}\Delta Bbmsn2$ strains by PCR. Lane M, Marker 5000. lane 3,4 and lane 1-4, four mutants. P, positive control with knockout vector as template. N, negative control with H_2O as template. (G) Real-time PCR analysis of knockout and overexpression strains. we choose *ΔBbPacCΔBbmsn2-42*, $BbPacC^{OE}\Delta Bbmsn2$*-3*, and $BbPacC^{OE}Bbmsn2^{OE}$*-31* for the further research.

Table S1 Primers in this study

**For RT-PCR analysis**	
PacC-F/R	TTGCGATCGCCATGTCGGCC//AAGGACTTGCCACAGAACTC
Msn2-F/R	ATGCTCGCAAGGCCTATCTG//GGCACTTCCAGAAGTGGACA
OpS3-F/R	CGGCTCTCCGAAACAATAAG//GTTGAAGCCAGTCAGGAAGG
OpS1-F/R	GCCGAAGGTGACCGTATT//GCGGGTTGATTCTGGACT
actin-F/R	GTCAAGTCATCACCATTGGC//GAGGAGCAATGATCTTGACC
**For mutant construction**	
PacC-LB1	CGGAATTCCTCACGCTGCAGCGAAACAG
PacC-LB2	GGACTAGTTGTCGGTTTAGATGTTAGCC
PacC-RB1	GCTCTAGAGCACAAACAACCTTAGCCTG
PacC-RB2	CCCAAGCTTACGTCGACGTCCATGGCATC
PacC-t1	GAGCCTGTCAGTGTACAAGGC
PacC-t2	CATCGGCATGTGTCTTGACGT
Msn2-LB1	CGGAATTCCACGAACGTCACATCACACC
Msn2-LB2	GGACTAGTAGGGGCGAGTATCGAGAGATCGAT
Msn2-RB1	GCTCTAGACAAGGACCTGTTTGACTCGG
Msn2-RB2	CCCAAGCTTCTTGTTATCAGAGGCGTCTTCAT
Msn2-t1	CGACCAGTCGACTCTCTTGGC
Msn2-t2	TGCTGGAGAGCTAGAGGGTGC
surTtrpC-R	AAGAAGGATTACCTCTAAACAAG
**For gene overexpression**	
PacC-O1	GCTCTAGAATGTCGCAACTCCCCGACAT
PacC-O2	GCTCTAGAATCAAGAACCGGGCACGGGGA
Msn2-O1	GCTCTAGAATGGAAGCTGCAATGCTGCA
Msn2-O2	GCTCTAGATTAGTCGGTGCGCTTGCGCT
**For yeast one-hybrid assay**	
PacC-42AD-F	CCGGAATTCATGTCGCAACTCCCCGACAT
PacC-42AD-R	CCGCTCGAGTCAAGAACCGGGCACGGGGA
*PacC*B1-F	AATTCAAAGCCAAGACTAAAGCCAAGACTAAAGCCAAGACTC
*PacC*B1-R	TCGAGAGTCTTGGCTTTAGTCTTGGCTTTAGTCTTGGCTTTG
*PacC*B2-F	AATTCGATCTTGGCAGGTACGCTTGGCAGGTGATCTTGGCAGGTACGCTTGGC AGGTC
*PacC*B2-R	TCGAGACCTGCCAAGCGTACCTGCCAAGATCACCTGCCAAGCGTACCTGCCA AGATCG
*PacC*B3-F	AATTCATCGCTTGGCATTGTATCGCTTGGCATTGTATCGCTTGGCATTGTC
*PacC*B3-R	TCGAGACAATGCCAAGCGATACAATGCCAAGCGATACAATGCCAAGCGATG
**For EMSA assay**	
PacC-F	AGATCTTGGCAGGTACGCTTGGCAGGTC
PacC-R	GACCTGCCAAGCGTACCTGCCAAGATCT
PaCompete-F	AGATCTTGGCAGGTACGCTTGGCAGGTC
PaCompete-R	GACCTGCCAAGCGTACCTGCCAAGATCT
msn2-F	AGTGGTGCCCCTAATGACGA
msn2-R	TCGTCATTAGGGGCACCACT

msCompete-F	AGTGGTGCCCCTAATGACGA
msCompete-R	TCGTCATTAGGGGCACCACT
N-PacC-F	CAACTCTTATCGCTTGGCATTGTGCTTTCAG
N-PacC-R	CTGAAAGCACAATGCCAAGCGATAAGAGTTG
cN-PacC-F	CAACTCTTATCGCTTGGCATTGTGCTTTCAG
cN-PacC-R	CTGAAAGCACAATGCCAAGCGATAAGAGTTG
